# Auditory temporal resolution and backward masking in musicians with absolute pitch

**DOI:** 10.3389/fnins.2023.1151776

**Published:** 2023-04-17

**Authors:** Carlos Alberto Leite Filho, Caroline Nunes Rocha-Muniz, Liliane Desgualdo Pereira, Eliane Schochat

**Affiliations:** ^1^Auditory Processing Lab, Department of Physical Therapy, Speech-Language Pathology and Occupational Therapy, School of Medicine, University of São Paulo, São Paulo, Brazil; ^2^Speech-Language Pathology Department, Santa Casa de São Paulo School of Medical Sciences, São Paulo, Brazil; ^3^Neuroaudiology Lab, Department of Speech Therapy, Paulista School of Medicine, Federal University of São Paulo, São Paulo, Brazil

**Keywords:** temporal resolution, backward masking, absolute pitch, auditory temporal processing, music, auditory perception, hearing tests

## Abstract

Among the many questions regarding the ability to effortlessly name musical notes without a reference, also known as absolute pitch, the neural processes by which this phenomenon operates are still a matter of debate. Although a perceptual subprocess is currently accepted by the literature, the participation of some aspects of auditory processing still needs to be determined. We conducted two experiments to investigate the relationship between absolute pitch and two aspects of auditory temporal processing, namely temporal resolution and backward masking. In the first experiment, musicians were organized into two groups according to the presence of absolute pitch, as determined by a pitch identification test, and compared regarding their performance in the Gaps-in-Noise test, a gap detection task for assessing temporal resolution. Despite the lack of statistically significant difference between the groups, the Gaps-in-Noise test measures were significant predictors of the measures for pitch naming precision, even after controlling for possible confounding variables. In the second experiment, another two groups of musicians with and without absolute pitch were submitted to the backward masking test, with no difference between the groups and no correlation between backward masking and absolute pitch measures. The results from both experiments suggest that only part of temporal processing is involved in absolute pitch, indicating that not all aspects of auditory perception are related to the perceptual subprocess. Possible explanations for these findings include the notable overlap of brain areas involved in both temporal resolution and absolute pitch, which is not present in the case of backward masking, and the relevance of temporal resolution to analyze the temporal fine structure of sound in pitch perception.

## 1. Introduction

Absolute pitch (AP) is commonly defined as the ability to identify the pitch of a musical note without external reference. Most humans identify musical pitches more relatively than absolutely. However, musicians with highly developed AP can do so in an absolute and effortless way, similar to the process of identifying colors (Takeuchi and Hulse, 1993; Deutsch, 2013).

AP presents a unique set of features that has intrigued scientists for over a century, like better pitch identification for piano tones over synthetic, violin, and voice tones (Miyazaki, 1989; Marvin and Brinkman, 2000; Schlemmer et al., 2005; Vanzella and Schellenberg, 2010) and discrepancies of pitch identification between octaves (Miyazaki, 1989). Besides that, AP shows numerous associations with non-musical phenomena, including more autistic traits among individuals with better pitch identification ability (Dohn et al., 2012), larger auditory working memory span in musicians with AP (Deutsch and Dooley, 2013), shared neural substrates between AP and synesthesia (Loui et al., 2012a), and higher occurrence of AP in speakers of tone languages (Deutsch et al., 2006, 2009).

The genesis of AP is still a matter of debate. The “critical period hypothesis” argues that AP emerges from early exposure to musical training during childhood. Evidence supporting this hypothesis shows that musicians with earlier onset of musical training also perform better at pitch identification tasks (Gregersen et al., 1999; Deutsch et al., 2006, 2009; Vanzella and Schellenberg, 2010; Leite et al., 2016), and young children outperform adults in pitch identification tasks after only 3 weeks of training (Russo et al., 2003). The “genetic hypothesis” states that AP is a genetically determined trait, and studies have pointed to genes associated with AP (Theusch et al., 2009; Gregersen et al., 2013) and to a higher prevalence of high-performance AP among twins in comparison to non-twins (Theusch and Gitschier, 2011; Bairnsfather et al., 2022a). Finally, although initially disregarded, the “practice hypothesis” gained attention from the scientific community with recent studies showing that, with intensive training, some adults are capable of reaching an extraordinarily high and long-lasting precision at identifying pitches, comparable to musicians with “naturally” high AP ability (Van Hedger et al., 2019; Wong et al., 2020a, 2020b). Considered alone, however, these hypotheses fail to fully explain the characteristics variance observed in AP, thus reinforcing the notion that this phenomenon most likely arises from the interaction between genetic and environmental factors (Zatorre, 2003; Bermudez and Zatorre, 2009a; Szyfter and Witt, 2020).

Whether AP is an all-or-none ability or a continuum is an unresolved issue. Researchers proposing that AP is an all-or-none ability have used different cut-off points in different parameters of pitch identification tasks, such as hit rate, reaction time, deviation from the original note, and the number of timbres tested. This results in pronounced methodological heterogeneity and difficulty comparing studies (Sergeant and Vraka, 2014; Germano et al., 2016; Bairnsfather et al., 2022b). Others reject that notion and place AP in the “continuum feature” category (Schellenberg and Trehub, 2003; Vitouch, 2003; Gußmack et al., 2006; Chavarria-Soley, 2016; Leite et al., 2016; Jakubowski et al., 2017).

The processes by which AP operates are also an open question. The cognitive model proposes that AP is based mainly on cognitive processes, namely long-term pitch memory and the association of these pitches to verbal labels (Levitin, 1994; Levitin and Rogers, 2005). On the other hand, the perceptual model presents AP as the product of a unique capability of mental categorization of musical notes. This phenomenon is a byproduct of improved auditory perception and its association with pitch labeling (Siegel, 1974; Zatorre, 2003).

The dual-stream model for auditory processing may be used to unify both mechanisms that seek to explain AP. In that sense, cognitive and perceptual processes, mediated by dorsal and ventral streams, respectively, act as complementary subprocesses on AP (Elmer et al., 2015; Leipold et al., 2019a).

The perceptual subprocess, or the role of auditory processing in AP, is very complex and needs further investigation. Many studies approach it through behavioral, electrophysiological, and neuroimaging techniques, sometimes with conflicting results (Kim and Knösche, 2017a).

From a behavioral perspective, evidence shows that pitch identification is more precise for iterated rippled noise, which provides temporal cues for pitch processing in the auditory pathway, than for narrow band noise, in which pitch information resides primarily on place cues (Fujisaki and Kashino, 2005). Another study showed that musicians with high precision AP were more efficient in identifying speech syllables in an automated, bottom-up task than musicians with low performance in a pitch identification task (Masataka, 2011). In a recent study, frequency resolution predicted performance at pitch labeling (Reis et al., 2021). However, other studies found no evidence of a correlation between AP and enhanced auditory skills, like temporal, spatial, and frequency resolutions (Fujisaki and Kashino, 2002; McKetton et al., 2019). Thus, among the many aspects involved in auditory perception, the relationship between temporal processing and AP still needs to be clarified.

Temporal processing refers to the perception of sound and its changes in a defined time domain, and since sound is essentially a physical event distributed in time, one can assume that temporal processing plays a role in most auditory processing skills (Phillips, 1999; Shinn, 2003).

Temporal resolution, one of the mechanisms involved in temporal processing, can be defined as the shortest time needed to discriminate between two auditory signals (Shinn, 2003). Traditionally, temporal resolution has been assessed through gap detection tasks, in which subjects must indicate whenever they perceive a short silent interval during a continuous auditory stimulus (Phillips et al., 1997; Phillips, 1999; Musiek and Chermak, 2015). Since detecting temporal gaps depends on representing such events along the auditory pathway, lower gap detection thresholds indicate higher temporal precision in neural activity (Phillips, 1999).

Backward masking is another auditory phenomenon linked to the temporal perception of sound (Shinn, 2003). In backward masking, the hearing threshold of a stimulus, usually a tone, is modified by a subsequent stimulus, usually a noise (Musiek and Chermak, 2015). Individuals with a central auditory nervous system (CANS) with higher temporal precision are expected to present lower masking efficiency of the noise over the tone previously presented.

Both temporal resolution and backward masking are linked to the processing of sounds by the CANS, especially the primary auditory cortex (Hartley et al., 2000; Musiek et al., 2005; MacDonald, 2011; Moore et al., 2011; Arnal et al., 2015; Eggermont, 2015), which is also closely associated with AP ability (Wengenroth et al., 2014; Kim and Knösche, 2016, 2017b; Brauchli et al., 2019; Burkhard et al., 2019; McKetton et al., 2019). Additionally, temporal processing is well established as a crucial component for pitch perception (Moore, 2008; Oxenham, 2012), further strengthening the hypothesis that temporal resolution and backward masking may play a role in AP.

In the present study, we conducted two experiments to verify whether musicians with AP showed distinct temporal processing, specifically in temporal resolution and backward masking domains. In the first experiment, musicians with and without AP completed a gap detection test. In the second experiment, we investigated the performance of musicians with and without AP in a backward masking test. We hypothesized that if auditory temporal processing plays a role in the perceptual subprocess of AP, then higher AP ability will be associated with enhanced performance in both experiments.

## 2. Experiment 1

In this experiment, we investigated the relationship between temporal resolution and AP by comparing musicians with and without AP (AP and Non-AP groups, respectively) regarding their performance at the Gaps-in-Noise (GIN) test, a clinical tool for assessing gap detection.

### 2.1. Materials and methods

#### 2.1.1. Subjects

The sample comprised 19 Brazilian young adult musicians without outer ear abnormalities and tonal auditory thresholds within normal limits [0–20 dB; 0.25–8 kHz (Loyd and Kaplan, 1978)) on both ears, as well as normal speech discrimination in silence (speech discrimination index ≥88% (Thornton and Raffin, 1978)]. All participants had no signs or symptoms of neurological, psychiatric, motor, language, learning, or auditory disorders. In addition, they were native speakers of Brazilian Portuguese, and none had contact with tone languages.

All subjects completed a pitch identification test described with greater detail elsewhere (Keenan et al., 2001; Loui et al., 2011). The test consisted of 13 sine wave stimuli with fundamental frequencies matching those of the 13 pitches between F#3 and F#4 in the equal-tempered Western scale. Each stimulus was presented binaurally four times, totaling 52 stimuli with a 2 s intertone time interval, in a pseudo-randomized order so that a specific stimulus only was presented again if all 12 other stimuli had already been presented. Testing was conducted in a silent room, and stimuli were presented at 50 dB SL. Stimuli were delivered through TDH-39 headphones (Telephonics Corp., Farmingdale, NY, United States) by a smartphone coupled to a PAC 200 two-channel portable audiometer (Auditec, São Paulo, SP, Brazil). Participants were asked to name each tone out loud according to the pitch chroma of the stimulus immediately after its presentation. The correct response rate was calculated considering only responses without deviation from the stimulus. Mean absolute deviation (MAD) (Bermudez and Zatorre, 2009b) was calculated to measure deviation from the target stimulus. Prior to the testing session, subjects underwent a training session consisting of one presentation of each stimulus. Musicians with a correct response rate of <50% at the training session did not undergo the testing session and were automatically assigned to the Non-AP group. Musicians were assigned to the AP group if they performed above the chance level (i.e., ≥ ten correct answers or 19.2%) with MAD <1.00 semitone. The correct response rate criterion was based on a statistical model, described in greater detail elsewhere (Sergeant and Vraka, 2014; Leite et al., 2016), in which a 99% confidence interval for the binomial mean was used as a cut-off point, while the MAD criterion was based on Loui et al. (2012b).

After the pitch identification test, the total sample was organized into two groups. AP group comprised nine musicians (four women, mean age: 22.89 ± 2.47 years old) with high performance at the pitch identification test (mean correct response rate = 84.62 ± 14.99%, range: 61.54–100%; MAD = 0.16 ± 0.15 semitones, range: 0.00–0.38 semitones). The Non-AP group comprised ten musicians (four women, mean age: 20.10 ± 3.00 years old). The mean age of onset of musical training was 8.22 ± 4.35 years-old and 10.60 ± 1.78 years old for AP and Non-AP groups, respectively. The mean duration of musical training was 14.33 ± 3.81 years and 6.90 ± 2.08 years for AP and Non-AP groups, respectively. All individuals from the AP group self-identified as AP possessors before testing, while none from the Non-AP group self-identified as such.

#### 2.1.2. Stimuli and procedures

The GIN test (Musiek et al., 2005) was designed as a clinically feasible assessment of auditory temporal resolution through the paradigm of gap detection. As a hearing test with high sensitivity and specificity for CANS dysfunctions (Filippini et al., 2020), the GIN test is a powerful tool for assessing central auditory function (Musiek, 2020).

Test stimuli consisted of 6 s segments of white noise with a 5 s interstimulus interval. Each segment contained 0 to 3 silent gaps presented randomly with durations of 2, 3, 4, 5, 6, 8, 10, 12, 15, or 20 ms. A complete assessment of one ear contained 29 to 36 stimuli, depending on the test track used, and 60 gaps divided evenly among the ten possibilities of gap duration.

The GIN test was delivered with the same equipment and conditions as the pitch identification test. Each subject had to listen to the stimuli and tap a pen at a table whenever they heard a gap. Both ears were assessed monaurally with different tracks for each ear (First ear: track 1, containing 35 stimuli; second ear: track 3, containing 29 stimuli). Ear differences were prevented by alternating the first ear tested of each individual. As such, half the sample had their right ears tested first, while the other half began with the left ear. Performance in each ear was analyzed with two measures: gap detection percentage (GDP), which represents the percentage of identified gaps from the total of 60 gaps presented, and gap detection threshold (GDT), or the shortest gap detected 4 out of 6 times with equal or higher detection rate for longer gaps. Each subject’s final performance was calculated by taking the average of the two ears’ GDP and GDT.

#### 2.1.3. Statistical analysis

Statistical analysis was conducted using SPSS Statistics for Windows, version 28.0 (IBM Corp., Armonk, NY, United States), and R 4.2.2 (R Core Team, 2022) with the *wBoot* (Weiss, 2016) and *robustbase* (Maechler et al., 2022) packages. Student’s independent-samples t-tests were used to compare groups against each other regarding GIN test measures. Correlation analyzes between the pitch identification and GIN tests were performed with Pearson’s correlation test. Since the data presented skewed distributions and the sample size was small, for the aforementioned analyzes, *p*-values were calculated using bias-corrected and accelerated 95% confidence intervals (BCa 95% CI) with 2000 bootstrap samples (Field, 2017). Finally, robust multiple linear regression models were built with GIN parameters serving as predictors for the pitch identification task measures while controlling for the onset age of musical training. Effect sizes (ES) were calculated using g (Hedges, 1981) and *f*^2^ (Cohen, 1992) coefficients. Statistical significance was set at 5% (*p* ≤ 0.05). Whenever multiple hypotheses were tested, p-values were adjusted with the Benjamini-Hochberg method to control for a false discovery rate of 5% (Benjamini and Hochberg, 1995), thus denoted as p_BH_. Due to the small sample size, which may reflect in non-significant *p*-values associated to low statistical power, the ESs were also taken into account for interpreting the significance of the results, with large ESs (following the criteria for small, medium, and large ES suggested by Cohen (1992)) as indicators of significant results.

### 2.2. Results

**Figure 1:**
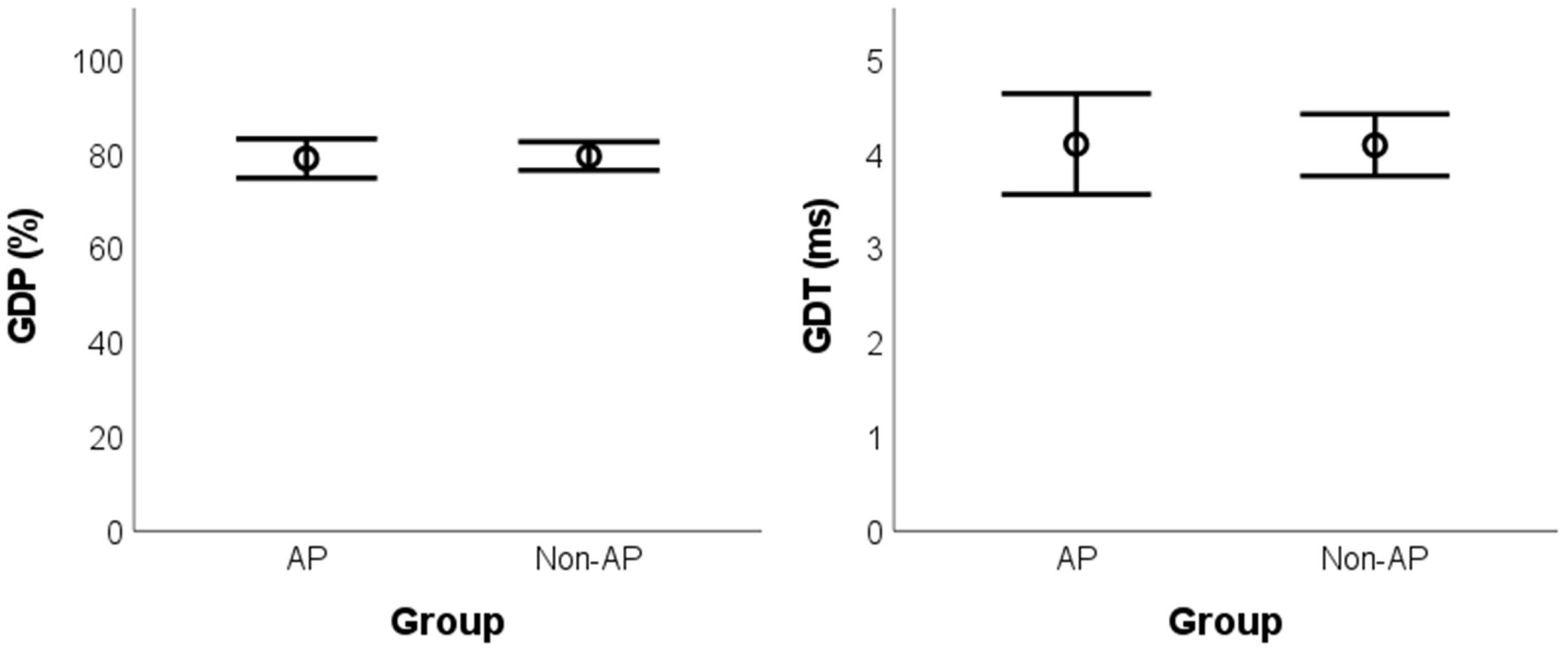
Mean values for the GIN parameters according to the group. GIN: Gaps-in-Noise; AP: absolute pitch; GDP: gap detection percentage; GDT: gap detection threshold.

For between-group comparisons, no statistically significant difference was found for GDP [*t*(17) = −0.229, p_BH_ = 0.967, g = 0.099, mean difference = −0.50%, BCa 95% CI = (−4.68, 3.18)] and GDT [*t*(17) = 0.042, p_BH_ = 0.967, g = 0.018, mean difference = 0.01 ms, BCa 95% CI = (−0.49, 0.59)] (Figure 1).

**Figure 2:**
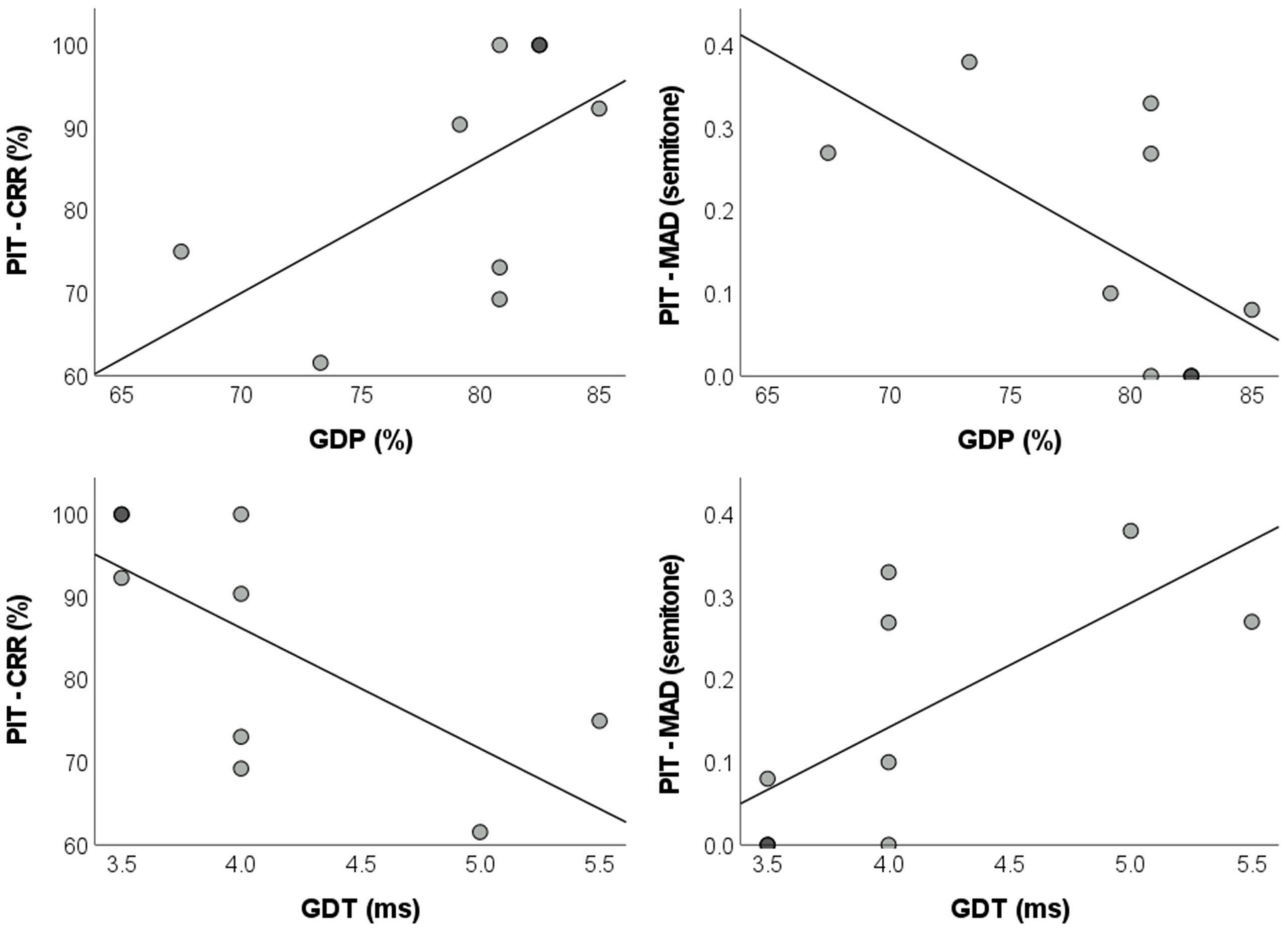
Dispersion plots with regression lines of the parameters for the pitch identification and GIN tests in the absolute pitch group. GIN: Gaps-in-Noise; GDP: gap detection percentage; GDT: gap detection threshold; PIT–CRR: pitch identification test–correct response rate; MAD: mean absolute deviation.

Statistically significant correlations between the pitch identification and the GIN tests parameters were found in the AP group. GDP was positively correlated to the correct response rate of the pitch identification test [*r* = 0.577, p_BH_ = 0.035, BCa 95% CI = (0.085, 0.842)] and negatively correlated to MAD [*r* = −0.587, p_BH_ = 0.035, BCa 95% CI = (−0.841, −0.081)]. As for GDT, a negative correlation with the correct response rate of the pitch identification was found [*r* = −0.679, p_BH_ = 0.028, BCa 95% CI = (−0.897, −0.253)], while a positive correlation with MAD was observed [*r* = 0.685, p_BH_ = 0.028, BCa 95% CI = (0.245, 0.897)]. Dispersion plots illustrating these analyzes are presented in Figure 2.

Robust regression analyzes without controlling for the onset age of musical training identified large effect sizes for all GIN measures as predictors of performance in the pitch identification test in the AP group. While GDP accounted for 30.00% of the variance for the correct response rate of the pitch identification test [adjusted *R*^2^ = 0.200, *F* (1,7) = 3.000, p_BH_ = 0.129, *f*^2^ = 0.429] and for 31.50% of the variance for the MAD [adjusted *R*^2^ = 0.217, *F*(1,7) = 3.219, p_BH_ = 0.129, *f*^2^ = 0.460], GDT was responsible for 42.80 and 44.20% of the variance observed in correct response rate (adjusted *R*^2^ = 0.346, *F*(1,7) = 5.238, p_BH_ = 0.112, *f*^2^ = 0.748) and MAD [adjusted *R*^2^ = 0.362, *F*(1,7) = 5.545, p_BH_ = 0.112, *f*^2^ = 0.792], respectively.

After controlling for the onset age of musical training, large effect sizes and significant or marginally significant *p*-values were observed for all GIN measures, with GDP accounting for 68.00% of the variance for the correct response rate of the pitch identification test [adjusted *R*^2^ = 0.526, *F*(1,6) = 16.721, p_BH_ = 0.017, *f*^2^ = 2.125] and for 65.90% of the variance for MAD [adjusted *R*^2^ = 0.495, *F*(1,6) = 14.921, p_BH_ = 0.017, *f*^2^ = 1.933]. GDT explained 45.20% of the variance for the correct response rate of the pitch identification test [adjusted *R*^2^ = 0.188, *F*(1,6) = 5.795, p_BH_ = 0.053, *f*^2^ = 0.825] and for 47.90% of the variance for MAD [adjusted *R*^2^ = 0.228, *F*(1,6) = 6.517, p_BH_ = 0.053, *f*^2^ = 0.919].

The complete robust multiple regression models are presented in Table 1. Significant regression coefficients were observed for all GIN test measures as predictors of the pitch identification test measures after controlling for the onset age of musical training. Notably, including the control variable did not lead to significant *R*^2^ increases. Moreover, a lack of statistical significance of the regression coefficients for the confounding variable was observed in all models (Supplementary Table S1).

**Table 1:** Robust multiple linear regression models of the measures of GIN test performance as predictors of performance at the pitch identification test while controlling for the onset age of musical training.

Model	Predictor	b [95% CI]	β [95% CI]	*p*
1. GDP as a predictor of the correct response rate for the pitch identification test	Constant	−232.68 [−539.01, 73.65]	--	0.112
	GDP	4.15 [0.06, 8.23]	1.50 [0.02, 2.98]	0.047*
	Onset age of musical training	−2.09 [−6.72, 2.54]	−0.61 [−1.95, 0.74]	0.312
2. GDP as a predictor of MAD	Constant	3.28 [0.27, 6.30]	--	0.037*
	GDP	−0.04 [−0.08, 0.00]	−1.45 [−2.89, 0.00]	0.047*
	Onset age of musical training	0.02 [−0.02, 0.07]	0.58 [−0.58, 2.03]	0.298
3. GDT as a predictor of the correct response rate for the pitch identification test	Constant	158.66 [104.02, 213.29]	--	< 0.001*
	GDT	−15.58 [−30.83, −0.33]	−0.73 [−1.44, −0.02]	0.047*
	Onset age of musical training	−1.19 [−3.21, 0.83]	−0.35 [−0.93, 0.24]	0.198
4. GDT as a predictor of MAD	Constant	−0.62 [−1.12, −0.11]	--	0.024*
	GDT	0.16 [0.02, 0.30]	0.75 [0.09, 1.40]	0.029*
	Onset age of musical training	0.01 [−0.01, 0.03]	0.29 [−0.29, 0.87]	0.165

Model 1: *R*^2^ = 0.756, adjusted *R*^2^ = 0.674, *F*(2,6) = 9.295, p_BH_ = 0.037; Model 2: *R*^2^ = 0.735, adjusted *R*^2^ = 0.647, F(2,6) =, p_BH_ = 0.037; Model 3: *R*^2^ = 0.532, adjusted *R*^2^ = 0.376, *F*(2,6) = 3.410, p_BH_ = 0.103; Model 4: *R*^2^ = 0.559, adjusted *R*^2^ = 0.411, F(2,6) = 3.803, p_BH_ = 0.103.

GIN: Gaps-in-Noise; GDP: gap detection percentage; GDT: gap detection threshold; 95% CI: 95% confidence interval; b: unstandardized coefficient; β: standardized coefficient; * and bold: statistically significant value at 5% level (*p* ≤ 0.05).

### 2.3. Discussion

Considering AP as an all-or-none ability, no relationship between pitch identification and temporal resolution was found due to the lack of significant differences between AP and Non-AP groups regarding the GIN test measures. However, from a continuum point-of-view, significant linear relationships were found between the measures of AP and GIN tests. Furthermore, GDP and GDT were significant predictors of AP measures, indicating that temporal resolution may influence AP.

To our knowledge, only one other study investigated gap detection in musicians with different levels of AP and found no evidence of enhanced temporal resolution compared to Non-AP musicians or non-musicians (Fujisaki and Kashino, 2002). However, comparisons between that research and the present investigation are limited due to relevant methodological discrepancies, mainly the criteria for assigning individuals to AP or Non-AP groups and the acoustic characteristics of the pitch identification test used in each experiment. In addition to this, Fujisaki and Kashino’s study lacks any correlational analysis between AP and gap detection scores.

One could argue that the lack of difference between groups in the present study and Fujisaki and Kashino’s investigation is evidence that the basic hearing ability of temporal resolution is unrelated to pitch identification, thus speaking against the participation of temporal processing in the perceptual subprocess of AP. In line with this argumentation is a neuroimaging study of gap detection tasks (Vaden et al., 2020) that reported a lack of activation of brain regions belonging to the ventral stream, such as the planum polare (Kim and Knösche, 2017b). However, considering that gap detection was able to predict pitch identification in the regression analyzes and that the approach to AP as a continuum trait is more suitable for the scoring method used in this investigation (Germano et al., 2021; Bairnsfather et al., 2022b), these results are most likely due to the inability of the arbitrary criteria used for the group assignment to generate clearly distinct groups.

Aware of such limitation, we conducted correlation and regression analyzes between the measures of pitch identification and GIN tests, showing that gap detection explained a high amount of the variance (between 20 and 36%, considering the values of adjusted R^2^ for the uncontrolled models) present in our sample’s pitch identification ability. These results align with other studies demonstrating that musicians with high-precision AP have improved basic hearing abilities, especially those related to temporal processing (Fujisaki and Kashino, 2005; Reis et al., 2021).

The influence of temporal resolution over AP precision corroborates the “perceptual subprocess hypothesis,” especially the component of absolute pitch categorization of this subprocess, in which unique hearing abilities lead to a specific categorization of musical notes, by indicating that auditory processing plays a role in defining the degree of precision of pitch naming ability (Siegel, 1974; Kim and Knösche, 2017a; Leipold et al., 2019a). Indeed, this relationship might reside in the crucial role of temporal processing in pitch perception due to the phase-locking attributes of neurons along the auditory pathway (Moore, 2008; Oxenham, 2012). A recent study verified that pitch naming is subserved by rapidly changing, transient processes at its initial stages (Ngan et al., 2023), further reinforcing that notion. Also, gap detection and pitch identification skills seem to share many neural substrates, as seen from neuroimaging and electrophysiological studies, such as bilateral Heschl gyri (Rupp et al., 2002, 2004; Heinrich et al., 2004; Diedler et al., 2007; Wengenroth et al., 2014; Kim and Knösche, 2016, 2017b; Rufener et al., 2017; Brauchli et al., 2019; Burkhard et al., 2019; McKetton et al., 2019; Vaden et al., 2020), bilateral superior temporal gyri (Heinrich et al., 2004; Wilson et al., 2009; Oechslin et al., 2010; Loui et al., 2012a,b; Schulze et al., 2013; Dohn et al., 2015; Vaden et al., 2020), bilateral inferior frontal gyri (Schulze et al., 2009; Wengenroth et al., 2014; Dohn et al., 2015; Leipold et al., 2019b; Vaden et al., 2020), right superior temporal sulcus (Schulze et al., 2009; Vaden et al., 2020), and right supramarginal gyrus (Schulze et al., 2013; Dohn et al., 2015; Vaden et al., 2020). It is worth noting that many of the cited structures are also part of the so-called “ventral stream,” which is heavily implicated in the perceptual subprocess of AP (Kim and Knösche, 2017a; Leipold et al., 2019a).

A possible limitation of this experiment is the use of a clinical test designed to identify CANS dysfunction to differentiate between two non-clinical groups. However, as previously seen in other studies, the GIN test can differentiate between groups with normal hearing and even identify differences between individuals with and without musical training (Ribeiro et al., 2015; Donai and Jennings, 2016).

## 3. Experiment 2

The second experiment intended to investigate the relationship between backward masking and AP. For this purpose, musicians with and without AP were compared regarding their performance in a novel tool for assessing backward masking.

### 3.1. Materials and methods

#### 3.1.1. Subjects

Fifteen young adults with similar audiological and medical characteristics to the volunteers of the first experiment comprised the sample of this experiment.

The pitch identification test used in this experiment was proposed by Leite et al. (2016). Test stimuli were 36 synthetic piano tones matching pitches between C3 and B5 in the equal-tempered Western scale. Each stimulus was presented twice in six blocks of 12 stimuli with 4.25 s of intertone time interval. In order to neutralize relative pitch-based responses, all tones presented tonal distance > one octave from the subsequent tone. TDH-39 headphones were used to present stimuli recorded in a smartphone coupled to a GSI-61 Audiometer (Grason-Stadler, Eden Prairie, MN, United States). The test was performed in a sound-attenuating booth with an intensity of presentation of 50 dB SL. Immediately after each note presentation, participants should mark the note (only pitch chroma) they heard in an answer sheet containing a 12-key musical keyboard illustration ranging from C to B. Correct response rate and MAD were calculated in the same way as the first experiment and musicians were assigned to the AP group following the same criteria of the previous experiment [correct response rate ≥ 20%, in accordance to the statistical approach (Sergeant and Vraka, 2014; Leite et al., 2016), and MAD <1.00 semitone(Loui et al., 2012b)].

All participants performed the pitch identification test, and two groups were formed according to their performances. AP group included eight musicians (three women, mean age: 25.88 ± 2.30 years old) with high performance at the pitch identification test (mean correct response = 88.54 ± 15.84%, range: 56.94–100%; MAD = 0.24 ± 0.34 semitones, range: 0.00–0.86 semitones). The Non-AP group included seven musicians (three women, mean age: 24.86 ± 3.18 years old) with low performance at the pitch identification test (mean correct response = 19.44 ± 5.32%, range: 13.89–29.17%; MAD = 2.37 ± 0.59 semitones, range: 1.54–2.99 semitones). The mean age of onset of musical training and mean duration of musical training for the AP group were 8.25 ± 3.99 years-old and 16.50 ± 5.26 years, respectively. For the Non-AP group, these values were 10.29 ± 2.69 years-old and 13.29 ± 4.61 years, respectively.

#### 3.1.2. Stimuli and procedures

For assessing backward masking, a backward masking test (BMT) designed for the clinical setting was used (Filippini et al., 2022). Unlike many other traditional psychoacoustic tests of backward masking, this test proposes a clinically feasible procedure that investigates the shortest inter-stimulus interval (ISI) needed for hearing a tone followed by a masking noise, and its results are comparable to those obtained with other tests reported in the literature (Filippini and Schochat, 2014; Filippini et al., 2022).

The BMT included two different stimuli: a 25 ms sine wave tone and a narrow-band 200 ms masking noise. The pure tone presentation level was set at 20 dB SL, and the signal-to-noise ratio was set at −20 dB. Each trial consisted of the monaural presentation of the sine wave tone followed by the presentation of the masking noise with an ISI of 400, 200, 100, 50, 30, 20, 10, or 0 ms between them. The complete evaluation of one ear consisted of six presentations of each ISI, totaling 48 trials plus 12 trials containing only the masking tone. Prior to the testing session, we conducted a training session with 12 trials with ISIs varying between 400 and 50 ms and three trials with masking noise only.

Participants were asked to listen carefully to the stimuli and press the response button whenever they heard the preceding tone. For each ear, the 60 trials were presented in different randomized orders. Half the sample tested their right ears first, while half had their left ears tested first to avoid ear differences due to the learning effect. Performance at the BMT was analyzed with two measures for each ear: percentage of correct responses (BMTP), which represents the percentage of correctly identified tones, and target sound detection threshold (BMTT), which is defined as the shortest ISI detected 4 out of 6 times with equal or higher detection rate for subsequent ISIs. The final performance for a given individual was considered as the average of the two ears’ BMTP and BMTT.

The equipment and conditions for the BMT were the same as those for the pitch identification test.

#### 3.1.3. Statistical analysis

Statistical procedures for this experiment included the same software, tests, bootstrapping technique, and significance criterion as used in Experiment 1. Since Experiment 2 was also conducted with a small sample, ESs were also considered for interpreting the results in the same fashion as Experiment 1.

### 3.2. Results

**Figure 3:**
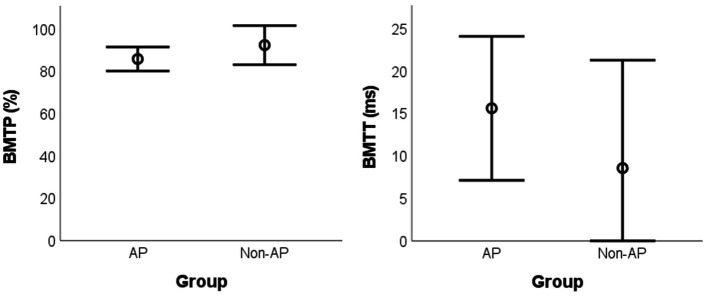
Mean values for the BMT parameters according to the group. BMT: Backward Masking Test; AP: absolute pitch; BMTP: BMT percentage of correct responses; BMTT: BMT target sound detection threshold.

The difference between groups was not significant for BMTP [*t*(13) = −1.993, p_BH_ = 0.194, g = 0.732, mean difference = −6.56%, BCa 95% CI = (−13.83, 2.40)] and for BMTT [*t*(13) = 1.506, p_BH_ = 0.194, g = 0.555, mean difference = 7.05 ms, BCa 95% CI = (−5.50, 18.32)] (Figure 3).

**Figure 4:**
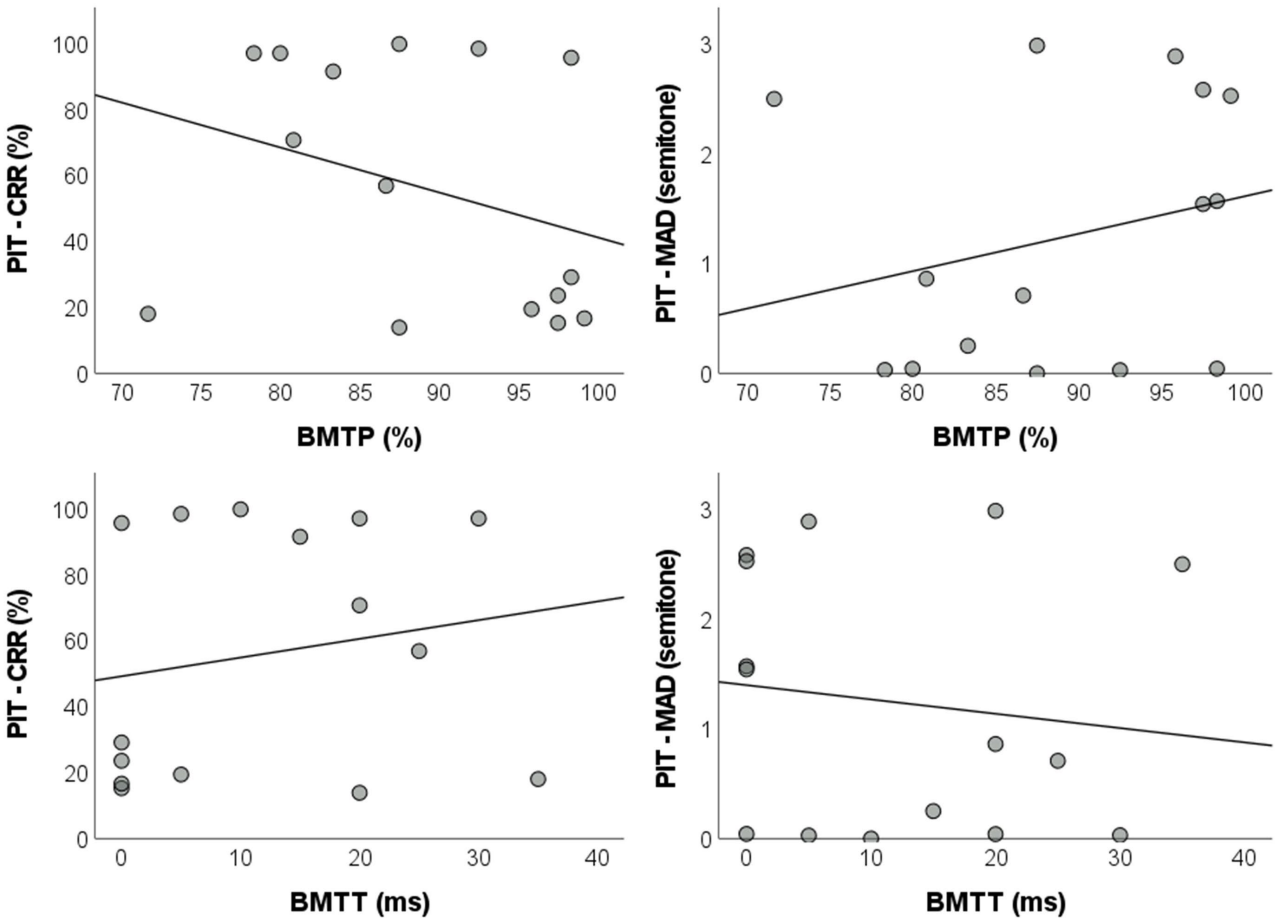
Dispersion plots with regression lines of the parameters for the pitch identification test and the BMT. BMT: backward masking test; BMTP: BMT percentage of correct responses; BMTT: BMT target sound detection threshold; PIT – CRR: pitch identification test – correct response rate; MAD: mean absolute deviation.

Correlation analyzes comprising the total sample of the study (AP + Non-AP groups, n = 15) revealed a lack of statistically significant linear correlations between BMTP and pitch identification test measures [correct response rate: *r* = −0.320, BCa 95% CI = (−0.777, 0.344), p_BH_ = 0.568; MAD: *r* = 0.253, BCa 95% CI = (−0.363, 0.708), p_BH_ = 0.568]. A similar scenario was observed between BMTT and the pitch identification test [correct response rate: *r* = 0.183, BCa 95% CI = (−0.409, 0.650), p_BH_ = 0.569; MAD: *r* = −0.132, BCa 95% CI = (−0.621, 0.568), p_BH_ = 0.652] (Figure 4). Due to the lack of significant correlations between the BMT and the pitch identification tests, no regression analyzes were carried out (Supplementary Table S2).

### 3.3. Discussion

The lack of statistical difference between groups and statistically significant correlations between the pitch identification test and the BMT suggests that backward masking is not related to the ability to name musical notes, either considering AP an all-or-none ability or a continuum trait.

These results agree with other studies which found no difference in the auditory perception of musicians with AP (Fujisaki and Kashino, 2002; McKetton et al., 2019). Although the data presented in Experiment 2 contradicts the “perceptual subprocess hypothesis” (Siegel, 1974; Fujisaki and Kashino, 2005; Kim and Knösche, 2017a; Leipold et al., 2019a; Reis et al., 2021) and even the results for Experiment 1, several reasons can be listed for this scenario.

First, the CANS is a highly complex set of structures; not all are involved in every aspect of auditory processing (Hackett, 2015; Pickles, 2015). In this sense, even though the neural basis of backward masking is still poorly understood (Mattingly et al., 2018), some findings suggest that AP and backward masking may have substantially different neural substrates. For example, while backward masking is related to a significant activation of the cingulate gyrus (van Dijk and Backes, 2003), this structure is not significantly activated during pitch identification by musicians with AP (Schulze et al., 2009). On the other hand, the activation of regions like the inferior frontal gyrus and the parietal lobule during AP-related tasks (Schulze et al., 2009; Wengenroth et al., 2014; Dohn et al., 2015; Leipold et al., 2019b) contrasts with the seemingly unremarkable activation of these areas during backward masking tasks (van Dijk and Backes, 2003). As such, even if the activity of structures like the temporal pole may suggest a neural overlap of backward masking and AP through the ventral stream (van Dijk and Backes, 2003), the cited dissociations may account for some of the results observed in Experiment 2.

Second, unlike the GIN test, and since it is a brand new auditory test, the potential of the BMT to differentiate between subgroups of individuals with normal auditory processing is yet to be investigated. Therefore, although the BMT can identify auditory processing disorders in a clinical context and presents a high correlation with other well-established temporal processing tests, including the GIN test (Filippini et al., 2022), the possibility that the tool cannot track subclinical, AP-related differences in temporal processing cannot be completely ruled out. However, it is worth noting that the absence of a significant ceiling effect in our sample speaks against this hypothesis.

Finally, a third possible explanation resides in the association between backward masking and cognitive abilities, such as short-term memory, attention, and executive function, as demonstrated by studies linking this auditory phenomenon to event-related auditory potentials, brain areas, and behavioral tasks that are known to take part in cognitive processes (Winkler and Näätänen, 1992; Winkler et al., 1993; Bazana and Stelmack, 2002; van Dijk and Backes, 2003; Strait et al., 2010; Parbery-Clark et al., 2011). Since cognitive factors were not strictly controlled in this experiment, a cognition-driven bias cannot be excluded entirely.

## 4. General discussion

Taken together, the results of both experiments provide further insights into the perceptual subprocess of AP. Although data from Experiment 1 suggests that temporal processing does play a role in pitch naming ability, the results from Experiment 2 are against this claim. Such discrepancies may reflect the discrete neural processes by which aspects of temporal processing operate and relate to other hearing abilities. In this context, some studies show a higher correlation coefficient between frequency discrimination and temporal acuity measured by gap detection (Jones et al., 2009; Gyldenkærne et al., 2014) than between frequency discrimination and the phenomenon of backward masking (Filippini et al., 2022), which may be an explanation for the apparently contradictory conclusions between experiments 1 and 2. Therefore, the results of the present research suggest that temporal resolution is involved in the perceptual subprocess of AP, while backward masking is not, implying that only some of the auditory temporal processing features are relevant to the assumption of unique categorization of musical notes present in the perceptual subprocess hypothesis (Siegel, 1974).

The present study also shows that treating AP as an all-or-none ability or a continuum trait may directly affect interpreting the results. In this sense, combining group and correlation analyzes in AP research may be a viable way to fully assess the phenomenon and overcome some of the methodological heterogeneity in the field.

A limitation worth noting for both experiments is the small sample size, which can be justified by the rare occurrence of high-precision AP, even in the musically trained population. We attempted to overcome this issue by using bootstrapping and robust methods to guarantee the validity of all statistical analyzes. Besides that, ESs, less prone to small sample bias, were also considered for interpreting the results.

The experiments were conducted in different moments and institutions. Consequently, a different pitch identification test, judged as less susceptible to relative pitch bias than the one used in Experiment 1, was used for Experiment 2. Even though this research topic lacks a definitive and valid test for determining the presence of absolute pitch, which implicates significative heterogeneity not only for our results but for other studies as well, we acknowledge that the differences between the pitch identification tests might impose limitations in comparing the results of both experiments. Another possible limitation in comparing the experiments is the absence of a precise mensuration of the absolute pitch ability in Non-AP musicians in Experiment 1 since subjects were assigned to the Non-AP group after a simple screening procedure during the training session of the pitch identification test. However, the AP group of Experiment 1 still yielded significative variance, with correct response rates ranging from 62 to 100%. Thus, we believe that the results of the correlation analyzes presented in this experiment are still noteworthy.

Although we demonstrated a link between temporal resolution and AP, further investigations must be made to elucidate the causal relationship between both, if any. In this sense, the impact of an auditory training program focused on temporal aspects on the pitch naming ability might be an interesting way to explore this question.

## 5. Conclusion

Better performance in the GIN test was a significant predictor of AP, indicating that temporal resolution influences pitch naming ability. On the other hand, no relationship was found between BMT and the pitch identification test, suggesting that temporal masking is not related to AP.

## Data availability statement

The original contributions presented in the study are included in the article/Supplementary material, further inquiries can be directed to the corresponding author.

## Ethics statement

The studies involving human participants were reviewed and approved by Federal University of São Paulo Research Ethics Committee and University of São Paulo School of Medicine Research Ethics Committee. The patients/participants provided their written informed consent to participate in this study.

## Author contributions

CLF contributed to study design, data collection, data management, data analysis, and writing and reviewing of the manuscript. CR-M contributed to data collection, data analysis, and reviewing of the manuscript. LP and ES contributed to study design, reviewing of the manuscript, and supervision of the study. All authors contributed to the article and approved the submitted version.

## Funding

This research was funded by São Paulo Research Foundation/Fapesp (grant 2019/13817–1) and National Council for Scientific and Technological Development (CNPq).

## Conflict of interest

The authors declare that the research was conducted in the absence of any commercial or financial relationships that could be construed as a potential conflict of interest.

## Publisher’s note

All claims expressed in this article are solely those of the authors and do not necessarily represent those of their affiliated organizations, or those of the publisher, the editors and the reviewers. Any product that may be evaluated in this article, or claim that may be made by its manufacturer, is not guaranteed or endorsed by the publisher.
